# Time to tackle clonorchiasis in China

**DOI:** 10.1186/2049-9957-2-4

**Published:** 2013-02-19

**Authors:** Men-Bao Qian, Ying-Dan Chen, Fei Yan

**Affiliations:** 1 National Institute of Parasitic Diseases, Chinese Center for Disease Control and Prevention; WHO Collaborative Center for Malaria, Schistosomiasis and Filariasis; Key Laboratory of Parasite and Vector Biology, Ministry of Health, Shanghai, People’s Republic of China; 2 School of Public Health, Fudan University, Shanghai, People’s Republic of China

**Keywords:** *Clonorchis sinensis*, Clonorchiasis, Hepatitis B virus, Liver cancer, Research

## Abstract

Recent publication of the global epidemiology of clonorchiasis and its relationship with cholangiocarcinoma in the journal of Infectious Diseases of Poverty has stressed the importance of *Clonorchis sinensis* infection. To further demonstrate its threat on public health, especially in China, comparisons between clonorchiasis and hepatitis B are made in terms of epidemiology, clinical symptoms and carcinogenicity, disability, as well as changing trends. Furthermore, major problems and prioritized researches are argued, from basic biology to intervention. Imbalance between the majority of infected population and the minority of researches in China urges for more work from Chinese scientists and international cooperation.

## Multilingual abstracts

Please see Additional file 1 for translations of the abstract into the six official working languages of the United Nations.

## Background

A review on the global epidemiology of clonorchiasis and its relationship with cholangiocarcinoma (CCA) was published in the journal of Infectious Diseases of Poverty on 25^th^ October 2012 [1]. A total of 15 million people are estimated to be infected with *Clonorchis sinensis* in East Asia and nearly 5,000 CCA cases attributed to this infection may occur annually in the coming decades. That article seeks to elucidate the situation and impact of clonorchiasis. However, some more deserve to be discussed, especially in China.

## Discussion

### Comparisons between clonorchiasis and hepatitis B in China

Clonorchiasis ranks among the top neglected tropical diseases [2]. Thus, comparing it with hepatitis B will promote revealing its threat on public health in China.

**Figure 1:**
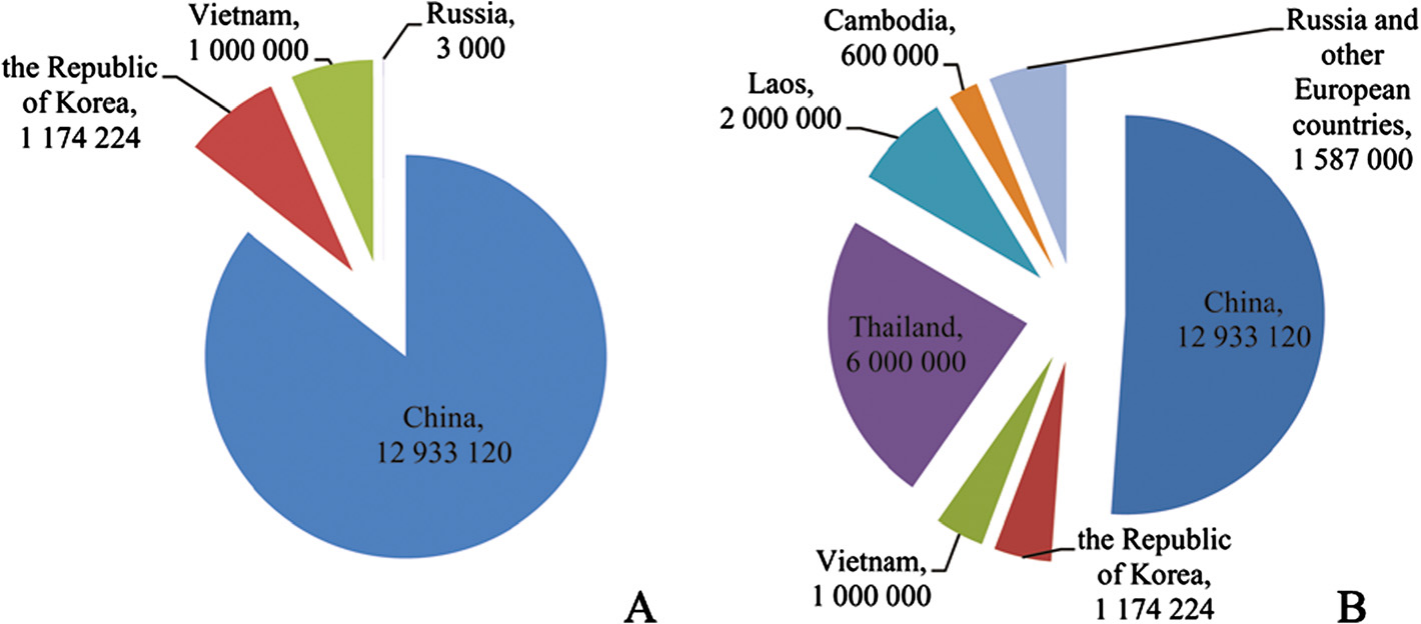
**Population with liver fluke infections globally. A**: Population with clonorchiasis. **B**: Population with clonorchiasis and opisthorchiasis.

**Figure 2:**
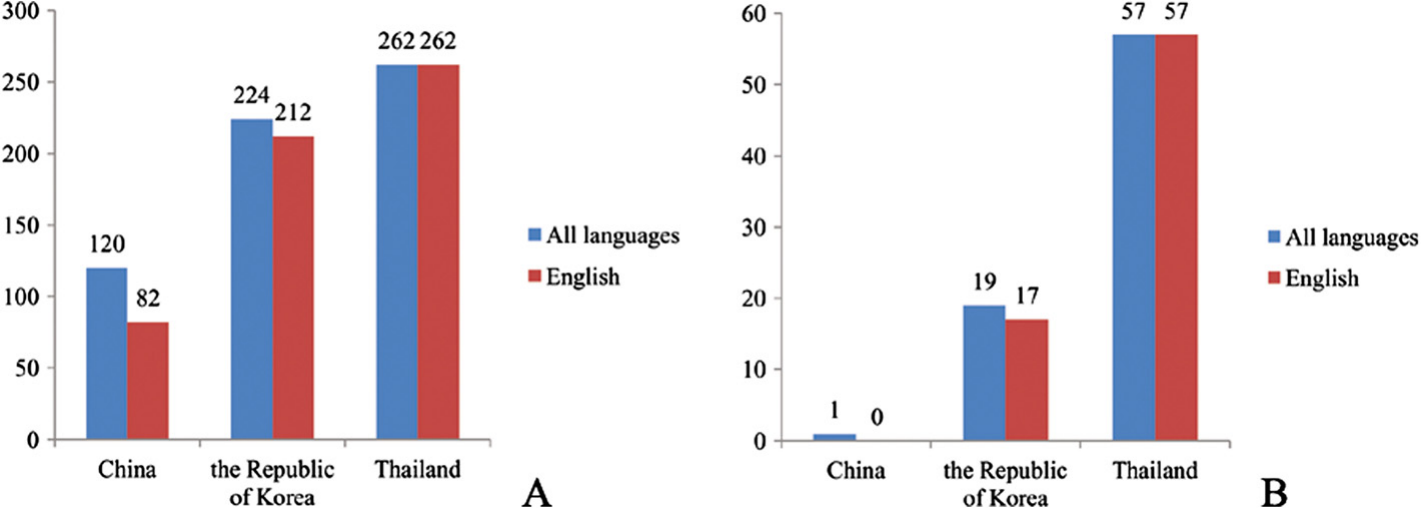
**Number of papers documented in Pubmed on liver flukes from China, the Republic of Korea and Thailand.** Searching strategy listed in Additional file 2. **A**: Papers focusing on liver flukes. **B**: Papers focusing on liver flukes and cholangiocarcinoma.

Firstly, most people infected with hepatitis B virus (HBV) distribute in China, and so do *C. sinensis* infections. A total population of 93 million is infected with HBV in China, which is about one quarter of the global number [3,4], while out of 15 million with *C. sinensis* infection globally, over 85% distributes in China (Figure 1A) [1]. Even after including another liver fluke infection, opisthorchiasis, the number in China is still over 50% (Figure 2B) [1,5,6]. Similar distribution characteristics in sexes and ages are shown in both infections, namely higher prevalence in males than in females and in adults than in children [1,7].

Secondly, as HBV mainly attacks the liver, *C. sinensis* infection also causes liver diseases, as well as biliary conditions. Although there are differences, some similarities may lead to misdiagnosis. Both may present non-specific symptoms or signs, such as fatigue, loss of appetite, fever, nausea, abdominal pain, jaundice and hepatomegaly [8,9]. What is most similar is that both can cause liver cancer [10,11]. Hepatocellular carcinoma and CCA are the two most common types of liver cancer [12]. HBV is a definite carcinogen to hepatocellular carcinoma, while *C. sinensis* is the one to CCA [10,11]. Meta-analyses captured a general odds ratio of 15.6 for HBV infection in causing hepatocellular carcinoma [13], and 4.5 for *C. sinensis* infection in causing CCA [1]. Inflammation is the crucial factor in the carcinogenesis of both agents [10]. As the carcinogenicity is associated with the virus load in HBV [14], it is relevant to the infection intensity in *Opisthorchis viverrini*-homologous to *C. sinensis*[15].

Thirdly, both HBV and *C. sinensis* infections cause significant disability. An average disability weight of 0.075 in *C. sinensis* infection is captured through community survey and model simulation [16], which is unexpectedly the same as that in hepatitis B [17].

Nevertheless, different changing trends appear. Although there is no availability of treatment to completely clear HBV, efficient vaccine and great efforts contribute to its obvious decline, especially in China [4,7,18]. Even though specific drug, namely praziquantel, is available, neglect and re-infection cause the significant increase in clonorchiasis [1,2].

Although there are fundamental differences in biology and some other aspects, comparisons above justify the public health importance of *C. sinensis* infection in China. Even though a relatively smaller population is infected with *C. sinensis* as compared with HBV nationally, the impact of clonorchiasis challenges hepatitis B in major endemic areas, namely the east part of China [1,19], where the former is becoming another killer of the liver and biliary health. The great success in controlling hepatitis B in China should provide valuable lessons for tackling clonorchiasis.

### More problems raised and more researches needed in China

Recently, the Disease Reference Group on Helminth Infections (DRG4) established by the Special Programme for Research and Training in Tropical Diseases ranked prioritized researches for the control and elimination of major human helminthiases, including clonorchiasis [20]. Indeed, some have also been pointed out directly or indirectly in our former article. 

Although three large-scale surveys involving clonorchiasis in China promote understanding the national situation and changes, epidemiology is only captured at the provincial level rather than at the county or lower level, which hampers the implementation of intervention measurements. Therefore, new methods, such as spatial techniques and modeling, deserve to be introduced to draw an epidemiological map for the disease [21,22]. Even though some epidemiological characteristics are known, social ecology and environmental determinants need to be elucidated further, such as the inherent drive for raw-fish-eating behavior, the cycle of infection-treatment-re-infection, factors involving in distributing and taking drug, roles of intermediate hosts, climate change [23], the property of zoonosis [24] and so on.

Whether other liver flukes and intestinal flukes are co-endemic in China challenges more accurate diagnosis, especially molecular methods [1,25]. Although two endemic zones have been classified [1] and some differences in biology have also been found [26], whether there exist differences in morbidity, especially in CCA, are also expected to be explored. It is already known that infection intensity is associated with carcinogenesis in *O. viverrini* infection [15], but corresponding studies in *C. sinensis* infection are not yet available, which causes unreasonably adopting the same OR in calculating CCA incidence in both sexes [1]. Furthermore, the impact of co-endemicity of *C. sinensis* and HBV infections on morbidity, especially on liver cancer in the east part of China, needs to be evaluated. Obviously, the establishment of tumor registry in China will promote related researches. Massive drug administration still counts on single drug, praziquantel [27], but no standardized principles of management are yet available. Additionally, tribendimidine, which shows promising efficiency against *C. sinensis* infection *in vivo*, *in vitro*, and in small field test, is expected to be further evaluated [28-30].

Even though most of the population infected with liver flukes distribute in China, corresponding researches are significantly less as compared with that in the Republic of Korea and Thailand, especially in CCA (Figure 2A and 2B, Additional file 2). The effort for controlling clonorchiasis in China will determine the global agenda for control and even elimination. Thus, more researches are anticipated from Chinese scientific workers. As it should be, international cooperation will be welcomed for tackling this problem [31].

## Summary

The comparability between clonorchiasis and hepatitis B in epidemiology, clinical symptoms and carcinogenicity and disability, and contrast in changing trends, justify the threat of clonorchiasis and the urgency for intervention in China. Prioritized researches covering topics from basic biology to intervention are expected, which will benefit the control and even final elimination of clonorchiasis. However, the imbalance between the majority of infected population and the minority of researches in China draws more challenges. Thus, more efforts and outputs are expected from Chinese scientists, as well as international cooperation.

## Competing interests

The authors declare that they have no competing interests.

## Authors’ contributions

MBQ, YDC and FY discussed jointly. MBQ developed the first draft, and all authors read and approved the final manuscript.

## Supplementary Material

Additional file 1 Multilingual abstracts in the six official working languages of the United Nations. Click here for file

Additional file 2 Analysis on the number of papers documented in Pubmed on liver flukes from China, the Republic of Korea and Thailand. Click here for file
