# Supplementary material for: Time to tackle clonorchiasis in China
Source: Infect Dis Poverty. 2013 Feb 19;2:4. doi: 10.1186/2049-9957-2-4 (PMC3707093; doi:10.1186/2049-9957-2-4)
Supplement: Additional file 1 — Multilingual abstracts in the six official working languages of the United Nations. [file 2049-9957-2-4-S1.pdf]

Translation of the abstract into the six official working languages of the United Nations

حان الوقت لمهاجمة داءُ مُتَفرِّعاتِ الخُصْيَة في الصين

مين باو كيان، ينغ دان تشين، فاي يان

الملخص
أكدت المنشورات الحديثة ذات الصلة بالوبائيات العالمية لداء متفرعات الخصية وعلاقتها بسرطانة الأقنية الصفراوية في أمراض الفقر المعدية، على أهمية عدوى مُتَفَرِّعُ الخُصْيَةِ الصِّينِيُّ. ولإظهار مدى تهديده للصحة العامة في الصين بشكل خاص، أجريت مقارنة بين داء متفرعات الخصية والتهاب الكبد الوبائي ب فيما يتعلق بالوبائيات والأعراض السريرية والسرطنة والعجز بالإضافة إلى تغيير الاتجاه. وعدا عن ذلك، هناك جدل حول المشاكل الرئيسية وتحديد أولويات البحوث ابتداء من البيولوجيا الأساسية وصولا إلى الابتكار. إن عدم التوازن بين إصابة معظم السكان بالعدوى وقلة البحوث المجراة في الصين تحث العلماء الصينيين على المزيد من العمل وعلى التعاون الدولي.

Translated from English version into Arabic by Lina SM, through

重视中国华支睾吸虫病的时候到了

钱门宝，陈颖丹，严非

摘要：发表于《Infectious Diseases of Poverty》首刊的“华支睾吸虫病全球流行情况及其与胆管癌的关系”一文强调了该病的重要性。此处，笔者从流行病学、临床症状和致癌性、疾病负担、变化趋势方面将其与乙肝进行比较，从而进一步阐述其对中国公共卫生的影响。此外，针对存在的主要问题和应该优先开展的研究也进行了探讨，涉及从基础研究到干预的各方面。中国感染此病人数虽然全球最多，但研究明显不足，因而有待中国科研工作者的努力和国际合作。

Translated from English version into Chinese by Qian Men-bao, through

Il est grand temps de s'occuper de la clonorchiase en Chine

Men-Bao Qian, Ying-Dan Chen, Fei Yan

Résumé: Une publication récente dans *Infectious Diseases of Poverty* sur l'épidémiologie mondiale de la clonorchiase et son lien avec le cholangiome malin a souligné l'importance de l'infection par *Clonorchis sinensis.* Pour illustrer la menace que constitue cette maladie pour la santé publique et tout particulièrement en Chine, des comparaisons ont été faites entre la chlonorchiase et l'hépatite B en termes d'épidémiologie, de symptômes cliniques, de cancérigènicité, de handicap et d'évolution. Cet article aborde en outre les principaux problèmes et les priorités de recherche du point de vue de la biologie de base, mais aussi de l'intervention. Le déséquilibre existant entre le grand nombre de personnes infectées et le nombre restreint de recherches réalisées en Chine met en évidence la nécessité d'un travail accru de la part des chercheurs chinois et d'une collaboration à l'échelle internationale.

Translated from English version into French by Marie Britton, through

TRANSLATORS
WITHOUT BORDERS

Пора найти меры против клонорхоза в Китае

Мен-Бао Чжен, Йинг-Дан Чен, Фей Ян

Содержание: Недавняя публикация в сфере мировой эпидемологии клонорхоза и его связи с холангиогенным раком в выпуске “Инфекционных заболеваний бедности” подчеркнула важность изучения инфекции китайской двуустки. Чтобы еще раз продемонстрировать, как это заболевание угрожает общественному здоровью, в особенности в Китае, был проведен сравнительный анализ между клонорхозом и гепатитом Б с точки зрения эпидемологии, клинических симптомов и канцерогенного воздействия, нарушения функций и тенденций к изменению. Кроме того, главные проблемы и приоритетные исследования обсуждаются как в области базовой биологии,

так и в сфере медицинского вмешательства. Неутешительное соотношение между числом инфецированного населения и количеством соответствующих исследований в Китае должно стимулировать более активную деятельность со стороны китайских ученых и их международнх партнеров.

Translated from English version into Russian by Elanorielle, through

TRANSLATORS
WITHOUT BORDERS

La lucha contra la clonorquiasis en China

´

Men-Bao Qian, Ying-Dan Chen y Fei Yan

Sinopsis: Una reciente publicación sobre la epidemiología de la clonorquiasis a nivel mundial y su relación con el colangiocarcinoma en enfermedades infecciosas derivadas de la pobreza ha subrayado la importancia de la infección por el parásito *Clonorchis sinensis.* Para reforzar la evidencia de que dicha infección representa una amenaza para la salud pública, especialmente en China, se comparan la clonorquiasis y la hepatitis B desde el punto de vista de su epidemiología, carcinogenicidad y síntomas clínicos, discapacidad y tendencia de cambio. Asimismo, se debaten las investigaciones prioritarias y sus principales problemas, desde la biología básica hasta la intervención. El desequilibrio que existe en China entre la gran cantidad de población infectada y la poca investigación que se lleva a cabo insta a la comunidad científica del país a realizar mayores esfuerzos en este campo y a la comunidad internacional a colaborar en ello.

Translated from English version into Spanish by Marta Callava Linares, through

TRANSLATORS
WITHOUT BORDERS
