# Supplementary material for: Time to tackle clonorchiasis in China
Source: Infect Dis Poverty. 2013 Feb 19;2:4. doi: 10.1186/2049-9957-2-4 (PMC3707093; doi:10.1186/2049-9957-2-4)
Supplement: Additional file 2 — Analysis on the number of papers documented in Pubmed on liver flukes from China, the Republic of Korea and Thailand. [file 2049-9957-2-4-S2.doc]

**Supplement 1:**

Analysis on the number of papers documented in Pubmed on liver flukes from China, the Republic of Korea and Thailand.

**Database:**

Pubmed

**Time limit:**

No

**Searching time:**

2012-11-1

**Searching strategy: as below**

| Search | Add to builder | Query | Items found | Time |
| --- | --- | --- | --- | --- |
| [#19](http://www.ncbi.nlm.nih.gov/pubmed/advanced) | [Add](http://www.ncbi.nlm.nih.gov/pubmed/advanced) | Search **(#15) AND english[Language]** | [57](http://www.ncbi.nlm.nih.gov/pubmed/?cmd=HistorySearch&querykey=19) | 08:26:57 |
| [#18](http://www.ncbi.nlm.nih.gov/pubmed/advanced) | [Add](http://www.ncbi.nlm.nih.gov/pubmed/advanced) | Search **(#14) AND english[Language]** | [17](http://www.ncbi.nlm.nih.gov/pubmed/?cmd=HistorySearch&querykey=18) | 08:26:09 |
| [#16](http://www.ncbi.nlm.nih.gov/pubmed/advanced) | [Add](http://www.ncbi.nlm.nih.gov/pubmed/advanced) | Search **(#13) AND english[Language]** | [0](http://www.ncbi.nlm.nih.gov/pubmed/?cmd=HistorySearch&querykey=16) | 08:25:29 |
| [#15](http://www.ncbi.nlm.nih.gov/pubmed/advanced) | [Add](http://www.ncbi.nlm.nih.gov/pubmed/advanced) | Search **(#12) AND Thailand[Affiliation]** | [57](http://www.ncbi.nlm.nih.gov/pubmed/?cmd=HistorySearch&querykey=15) | 08:24:45 |
| [#14](http://www.ncbi.nlm.nih.gov/pubmed/advanced) | [Add](http://www.ncbi.nlm.nih.gov/pubmed/advanced) | Search **(#12) AND Korea[Affiliation]** | [19](http://www.ncbi.nlm.nih.gov/pubmed/?cmd=HistorySearch&querykey=14) | 08:24:20 |
| [#13](http://www.ncbi.nlm.nih.gov/pubmed/advanced) | [Add](http://www.ncbi.nlm.nih.gov/pubmed/advanced) | Search **(#12) AND China[Affiliation]** | [1](http://www.ncbi.nlm.nih.gov/pubmed/?cmd=HistorySearch&querykey=13) | 08:23:08 |
| [#12](http://www.ncbi.nlm.nih.gov/pubmed/advanced) | [Add](http://www.ncbi.nlm.nih.gov/pubmed/advanced) | Search **(#10) OR #11** | [105](http://www.ncbi.nlm.nih.gov/pubmed/?cmd=HistorySearch&querykey=12) | 08:22:43 |
| [#11](http://www.ncbi.nlm.nih.gov/pubmed/advanced) | [Add](http://www.ncbi.nlm.nih.gov/pubmed/advanced) | Search **(opisthorchi*[MeSH Terms]) AND cholangiocarcinoma[MeSH Terms]** | [96](http://www.ncbi.nlm.nih.gov/pubmed/?cmd=HistorySearch&querykey=11) | 08:22:19 |
| [#10](http://www.ncbi.nlm.nih.gov/pubmed/advanced) | [Add](http://www.ncbi.nlm.nih.gov/pubmed/advanced) | Search **(clonorchi*[MeSH Terms]) AND cholangiocarcinoma[MeSH Terms]** | [27](http://www.ncbi.nlm.nih.gov/pubmed/?cmd=HistorySearch&querykey=10) | 08:21:37 |
| [#9](http://www.ncbi.nlm.nih.gov/pubmed/advanced) | [Add](http://www.ncbi.nlm.nih.gov/pubmed/advanced) | Search **(#6) AND english[Language]** | [262](http://www.ncbi.nlm.nih.gov/pubmed/?cmd=HistorySearch&querykey=9) | 08:20:28 |
| [#8](http://www.ncbi.nlm.nih.gov/pubmed/advanced) | [Add](http://www.ncbi.nlm.nih.gov/pubmed/advanced) | Search **(#5) AND english[Language]** | [212](http://www.ncbi.nlm.nih.gov/pubmed/?cmd=HistorySearch&querykey=8) | 08:19:52 |
| [#7](http://www.ncbi.nlm.nih.gov/pubmed/advanced) | [Add](http://www.ncbi.nlm.nih.gov/pubmed/advanced) | Search **(#4) AND english[Language]** | [82](http://www.ncbi.nlm.nih.gov/pubmed/?cmd=HistorySearch&querykey=7) | 08:19:25 |
| [#6](http://www.ncbi.nlm.nih.gov/pubmed/advanced) | [Add](http://www.ncbi.nlm.nih.gov/pubmed/advanced) | Search **(#3) AND Thailand[Affiliation]** | [262](http://www.ncbi.nlm.nih.gov/pubmed/?cmd=HistorySearch&querykey=6) | 08:18:00 |
| [#5](http://www.ncbi.nlm.nih.gov/pubmed/advanced) | [Add](http://www.ncbi.nlm.nih.gov/pubmed/advanced) | Search **(#3) AND Korea[Affiliation]** | [224](http://www.ncbi.nlm.nih.gov/pubmed/?cmd=HistorySearch&querykey=5) | 08:17:33 |
| [#4](http://www.ncbi.nlm.nih.gov/pubmed/advanced) | [Add](http://www.ncbi.nlm.nih.gov/pubmed/advanced) | Search **(#3) AND China[Affiliation]** | [120](http://www.ncbi.nlm.nih.gov/pubmed/?cmd=HistorySearch&querykey=4) | 08:17:11 |
| [#3](http://www.ncbi.nlm.nih.gov/pubmed/advanced) | [Add](http://www.ncbi.nlm.nih.gov/pubmed/advanced) | Search **(clonorchi*[MeSH Terms]) OR opisthorchi*[MeSH Terms]** | [2173](http://www.ncbi.nlm.nih.gov/pubmed/?cmd=HistorySearch&querykey=3) | 08:16:30 |
| [#2](http://www.ncbi.nlm.nih.gov/pubmed/advanced) | [Add](http://www.ncbi.nlm.nih.gov/pubmed/advanced) | Search **opisthorchi*[MeSH Terms]** | [1876](http://www.ncbi.nlm.nih.gov/pubmed/?cmd=HistorySearch&querykey=2) | 08:16:01 |
| [#1](http://www.ncbi.nlm.nih.gov/pubmed/advanced) | [Add](http://www.ncbi.nlm.nih.gov/pubmed/advanced) | Search **clonorchi*[MeSH Terms]** | [665](http://www.ncbi.nlm.nih.gov/pubmed/?cmd=HistorySearch&querykey=1) | 08:15:42 |

**Note:** To avoid including too many papers, which are less relevant, Mesh Terms were used. Thus, some relevant papers were unfortunately missed. However, that should not severely change the results.
